# Olfactory mucosal mesenchymal stem cell-derived exosome Lnc A2M-AS1 ameliorates oxidative stress by regulating TP53INP1-mediated mitochondrial autophagy through interacting with IGF2BP1 in Parkinson’s diseases

**DOI:** 10.1007/s10565-025-10009-7

**Published:** 2025-03-20

**Authors:** Jiangshan Zhang, Chuang Wang, Guoshuai Yang, Yanhui Zhou, Dan Hou, Ying Xia

**Affiliations:** 1 Department of Neurology, Central South University Xiangya School of Medicine Affiliated Haikou Hospital, Haikou, 570208 Hainan Province People’s Republic of China; 2 Department of Neurosurgery, Central South University Xiangya School of Medicine Affiliated Haikou Hospital, No.43, Renmin Avenue, Meilan District, Haikou, 570208 Hainan Province People’s Republic of China

**Keywords:** Parkinson’s Disease, Olfactory Mucosa Mesenchymal Stem Cell, Exosome, Mitophagy, Oxidative Stress, Lnc A2M-AS1

## Abstract

**Background:**

Exosome Lnc A2M-AS1 from olfactory mucosa mesenchymal stem cells (OM-MSCs) can ameliorate oxidative stress by improving mitophagy in cardiomuscular cells; however, it remains unclear whether this effect exists in the brain tissues of patients with Parkinson’s disease (PD).

**Methods:**

OM-MSC–Exosomes were isolated and verified based on morphology and specific biomarkers. The effects of OM-MSC-Exo on mitochondrial autophagy, oxidative stress, and lncRNA A2M-AS1 were detected in MPP^+^-treated HT22 cells. The effects of OM-MSC-Exos on mitochondrial autophagy and oxidative stress were detected in an MPTP-induced Parkinson's disease (PD) model in C57BL/6 mice. The interaction between IGF2BP1, A2M-AS1, and TP53INP1 was assessed via RNA pull-down/RNA Immunoprecipitation and RNA stability assays. The effects of lnc A2M-AS1 on IGF2BP1/TP53INP1-mediated mitochondrial autophagy and oxidative stress were verified in MPP^+^-treated HT22 cells and MPTP-induced PD mouse models.

**Results:**

Exosomes isolated from olfactory mucosa mesenchymal stem cells were found to be rich in Lnc A2M-AS1. Lnc A2M-AS1 was proved to be able to ameliorate oxidative stress induced by MPP^+^ in HT22 cells. lncRNA A2M-AS1 regulates oxidative stress by enhancing mitophagy in HT22 cells. In addition, lncRNA A2M-AS1 induced mitophagy through TP53INP1 and mediated TP53INP1 expression by binding to IGF2BP1. Furthermore, OM-MSC-Exo and Lnc A2M-AS1 treatment improved symptoms and ameliorated oxidative stress in MPTP-induced PD mouse models.

**Conclusion:**

Collectively, lncRNA A2M-AS1 from OM-MSC-derived exosomes regulates TP53INP1 expression by targeting IGF2BP1 to induce mitophagy and ameliorate oxidative stress. OM-MSC-derived exosomes could potentially serve as promising candidates for new treatment methods for PD.

**Graphical Abstract:**

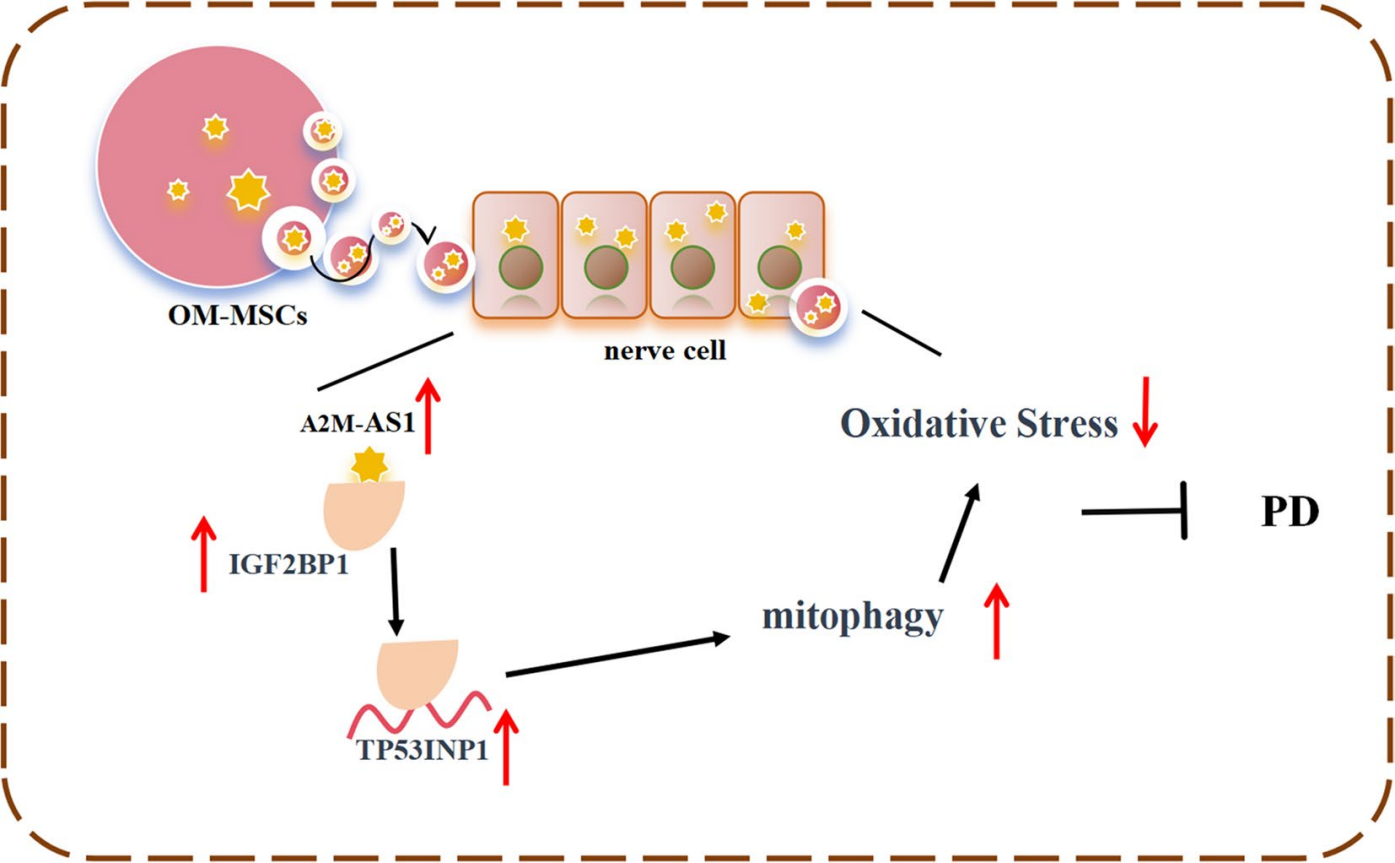

## Introduction

Parkinson’s disease (PD) is the second most common neurodegenerative disease worldwide. It has a relatively high rate of mortality and disability and is much more common among the elderly. In China there are approximately 13.7% senile individuals suffer from this disease (Hu and Xu 2023; Balestrino and Schapira 2020). The major pathological changes in PD are progressive degeneration and loss of dopaminergic neurons in the substantia nigra, and the formation of Lewy bodies. This change usually initiates from the dorsal motor nucleus of the glossopharyngeal and vagus nerves, and the anterior olfactory nucleus (Balestrino and Schapira 2020; Braak et al. 2003; Marsden 1990). The major symptoms of PD include dyskinesia, such as resting trembling, and other non-motor symptoms (Balestrino and Schapira 2020; Marsden 1990; Wakabayashi et al. 2013). Currently, the treatments in practice mainly include L-Dopa, a dopamine receptor agonist, and physical treatments such as deep brain stimulation (DBS), but these approaches mainly focus on improving symptoms and contribute poorly to preventing disease progression (Balestrino and Schapira 2020; Fox et al. 2011; Chiken and Nambu 2016).

Dysfunction in mitophagy and oxidative stress play essential roles in the pathology of PD (Geisler et al. 2010; Trinh and Farrer 2013). Studies have revealed that mediation in mitophagy could reduce oxidative stress damage in hippocampal neurons of rats with PD, and inducing mitophagy could protect neurons from neurotoxicity induced by 1-methyl-4-phenylpyridinium (MPP +) induced oxidative stress (Li et al. 2023; Williamson et al. 2023). Thus, it might be a potential novel direction in PD treatment to induce mitophagy to enhance the clearance of damaged mitochondria and to inhibit neuronal damage.

Mesenchymal Stem Cells (MSCs) are easy to access and can be used for autografts without immune rejection or complex ethical issues (Andrzejewska et al. 2021; Vizoso et al. xxxx; Heris et al. 2022). Li et al. and Al-Kharboosh et al. showed that stem cells and their secretomes could restore dopamine levels in patients with PD. For example, Li et al. showed that exomes of miR-188-3p from fat MSCs directly targeted genes related to PD pathology to inhibit autophagy and pyroptosis (Li et al. 2021; Al-Kharboosh et al. 2022). A series of studies based on MSCs targeting PD have already achieved good results in animal models (Mendes-Pinheiro et al. xxxx; Lei et al. 2022; Mendes-Pinheiro et al. 2019). Olfactory mucosa mesenchymal stem cells (OM-MSC) are stem cells with multi-differentiation potential that exist in the olfactory mucosa. It offers a new cell source for the treatment of degenerative neurological diseases, and plays a therapeutic role in nerve repair and functional reconstruction. Simorgh et al. and Alizadeh et al. have shown that olfactory mucosa stem cells are potent candidate cells for the treatment of PD (Simorgh et al. 2019; Alizadeh et al. 2019).

Long noncoding RNA (lncRNAs) are involved in various pathological and physiological processes. MSC-derived exosome Lnc A2M-AS1 alleviates apoptosis and oxidative stress in Cardiomyocytes (Yu et al. 2023). Li et al. found that A2M-AS1 is highly expressed in Alzheimer’s disease, indicating that this exosome may contribute to neurological degenerative diseases (Li et al. 2022), but its expression and role in PD has not yet been explored.

Recent studies have found that TP53INP1 plays a role in mitophagy to protect neurons under PD-related stress conditions such as oxidative stresses, and its deficiency leads to impairment of mitophagy (Seillier et al. 2015; Dinh et al. 2021; Wang et al. 2025). Dinh et al. proved that TP53INP1 deficiency triggers additional loss of substantia nigral dopaminergic neurons, exacerbating motor deficits and nigral dopaminergic neurodegeneration in α-syn overexpressing mice (Dinh et al. 2021). However, the role of Lnc A2M-AS1 in TP53INP1 remains unclear. Multiple studies have shown that lncRNAs can mediate downstream mRNA by interacting with RNA-binding proteins (Statello et al. 2021). In the present study, bioinformatic analysis that Lnc A2M-AS1could bind with IGF2BP1, IGF2BP1, and TP53INP1 mRNA, suggesting that Lnc A2M-AS1 mediates the stability and expression of IGF2BP1.

In this study, we investigated the potential protective role of lncRNA A2M-AS1 in OM-MSC-Exos using in vivo and in vitro models of PD. We hypothesized that OM-MSC-derived exosomes, Lnc A2M-AS1, regulate TP53INP1-mediated mitophagy by interacting with IGF2BP1 to reduce oxidative stress and improve the condition of Parkinson’s disease.

## Methods

### Patients and samples resources

This study was conducted under the guidance of the Ethics Committee of the Haikou People's Hospital (ethics approval no. 2024–89). All animal experiments were conducted in strict compliance with the IACUC guidelines.

### Cell culture

HT22 cells were purchased from Sigma-Aldrich and cultured in accordance with the manufacturer’s instructions. OM-MSCs were collected and cultured from the olfactory mucosa of mice. In this study, we collected samples from the root of the mouse middle turbinate and washed the samples with an antibiotic–antimycotic solution (Invitrogen, CA). The samples were cut into pieces with a length of 2 mm and thickness ranging from 300 to 400 μm. The sample pieces were placed in a dish containing Dulbecco’s modified Eagle’s medium (DMEM; Invitrogen, CA) containing 10% fetal bovine serum (FBS). The tissue slides were cultured at 37 °C and 5% CO_2_. The medium was changed every three days during the culture process.

### Isolation and identification of OM-MSCs exosome

The Exosomes were isolated from the OM-MSCs medium through ultracentrifugation using previously reported methods (Xun et al. 2020; Théry et al. xxxx). FBS was depleted with exosomes by ultracentrifugation at 100,000 × g for 18 h, and the supernatant was filtered with a 0.22 μm syringe filter (Millipore, USA). The 90% confluent OM-MCS cultures were washed twice with PBS and incubated in growth medium containing DMEM/F12 supplemented with 10% exosome-depleted FBS for two days. After that, cells were removed from conditioned medium (CM) together with dead cells and cells fractured under 300 g/10 min, 2000 g/10 min, and 10,000 g/30 min, respectively, at 4 °C. Then, the CM was filtered with a 0.22 μm syringe filter. The supernatant was first centrifuged at 100,000 g for 70 min at 4 °C using an SW32Ti rotor (Beckman Coulter, Netherlands). The exosome-containing pellet was washed with PBS and centrifuged at 100,000 g for 70 min. Finally, the pellet was resuspended in PBS (0.1 mL. The protein concentration was measured using a BCA kit (Beyotime, China).

### Cell transfection

HT22 cells were cultured in 6-well plates until attachment. To inhibit lncRNA A2M-AS1, TP53INP1, or IGF2BP1, short hairpin RNA (shRNA) against the corresponding genes, and noncoding shRNA (sh-NC) were obtained from GeneCopoecia (Guangzhou, China). In addition, Lnc A2M-AS1, TP53INP1, or IGF2BP1 sequences were inserted into the pcDNA3.1, vector (termed oe-Lnc A2M-AS1, oe-TP53INP1, or oe-IGF2BP1). An empty pcDNA3.1 vector (oe-NC) as a control was obtained from RiboBio (Guangzhou, China). All plasmids were transfected into HT22 cells using Lipofectamine 3000 (Invitrogen, USA) according to the manufacturer’s instructions, followed by co-culture with OM-MSCs derived exosomes as an experimental group or medium as a control. The corresponding cells were used for further experiments after 48 h of incubation.

### Ingestion of PKH67-labeled exosomes by recipient HT22 cells

The Exosome pellets were isolated as described above. The pellets were labeled with PKH67(Merck, United States) according to a previously described protocol (Pužar Dominkuš et al. 2018). The cells were then placed on an eight-well chamber slide. After 24 h incubation for quantitation, 8 μg of labeled exosomes was added to the media of HT22 cells and incubated together at 37 °C for 24 h. HT22 cells added with same volume of exosome-free medium were used as blank controls. The cells of the experiment group and the control group were fixed with 4% paraformaldehyde over a period of 20 min and stained with DAPI. Next, the uptake of exosomes was observed and evaluated using confocal microscopy.

### qPCR analysis

Total RNA was isolated using a RNeasy Mini Kit (Qiagen, Hilden, Germany). mRNA was extracted from total RNA using the Dynabeads mRNA DIRECT kit (61,006, Thermo Fisher Scientific) according to the manufacturer’s protocol. cDNA was synthesized from mRNA using TRIzol reagent (Invitrogen, USA) according to the manufacturer’s instructions. The PCR process was then conducted using the virous primers purchased from OriGENE company (USA) for corresponding genes including LC3, A2M-AS1 and TP53INP1 with SYBR Green PCR Master Mix kit (Takara, Japan) and 0.5 μL of DNA template under the instruction given by the manufacturer. Then the mixture was put into thermal cycling under these conditions: 95 °C for 3 min following by 95 °C for 30 s, 52 °C for 30 s and 72 °C for 50 s for 30 cycles. To quantify the expression level, the relative mRNA expression levels were defined with the fluorescence intensity observed.

### Western blotting

The Western blotting was performed according to classic instructions. The cells were collected, and total protein was extracted with RIPA lysis buffer (Thermo Fisher Scientific, USA). The quantity of total protein was measured using the Pierce BCA Protein Assay Kit (Thermofisher, China). Proteins were extracted from the medium and quantified using the same method. Then, 150 μL of the lysate protein mixture was mixed with 50 μL of 4 × loading buffer and boiled for 5 min. Total protein at Equal amounts were separated by 10% SDS (sodium dodecyl sulfate–polyacrylamide) gel electrophoresis and transferred onto polyvinylidene difluoride membranes (Beyotime, China). The membranes were immunoblotted using the following primary antibodies: CD9(1:1000, ab307085, Abcam), CD63(1:1000, ab315108, Abcam), calnexin (1:1000, ab22595, Abcam), LC3(1:2000, ab192890, Abcam), p62(1:10,000, ab109012, Abcam), COX IV (1:2000, ab202554, Abcam), TP53INP1(1:2000, ab202026, Abcam), IGF2BP1(1:1000, ab290736, Abcam), and TH (1:5000, ab137869, Abcam). After incubation at 4 °C for 8 h, the membranes were blocked with skimmed milk at a percentage of 5%. The secondary antibodies linked to horseradish peroxidase (Beyotime Institute of Biotechnology) were applied to the membrane, and the protein bands were visualized using ECL reagent (Thermo Fisher Scientific, USA) using a chemiluminescence system (Bio-Rad, USA). Total protein was semiquantified based on the ratio of GAPDH for each protein on each gel. In the present study, ImageJ software (1.53 version, United States) was used to quantify the results of Western Blotting.

### RNA Pull-Down and RNA immunoprecipitation (RIP) assay

RNA pull-down analysis was conducted as previous protocol described (Chopra et al. xxxx). the RNA–protein complex was immunoprecipitated using streptavidin magnetic beads (MedChemExpress, United States). The complex was then separated for PCR and western blotting. RIP was performed using an RIP kit (Merck, United States), according to the manufacturer’s instructions. RNA was purified using TRIzol (Thermo Fisher, United States) for qPCR analysis.

### RNA stability test

The Cells transfected with oe-A2M-AS1 and/or sh-IGF2BP1(short hairpin RNA) were treated with actinomycin D(ActD) (Merck, United States) at a dose of 5 μg/mL for 0, 3, and 6 h to inhibit transcription. The cells were then subjected to qPCR to measure level of TP53INP1 mRNA for the evaluation of the stability of TP53INP1 mRNA.

### Animal model

Neurotoxity was induced in C57BL/6 mice at an age of 10–12 weeks by giving MPTP through intraperitoneal injection at a dose of 20 mg/kg/d for 14 days to establish PD animal models. Mice injected with saline of the same dose during the same period were set as blank control.

To examine the inhibition effect of OM-MSC and the mechanism behind this, other two experiment groups were set with PD mice models injected OM-MSC-Exosomes and sh-Lnc A2M-AS1 together were set as other two experiment groups. Mice in each experimental group received 1.4 × 10 (Williamson et al. 2023) nanoparticles of OM-MSC exosomes, which were extracted in previous steps, with or without sh-Lnc A2M-AS1 nanoparticles of same dose, respectively, through stereotactic injection into the right lateral ventricle one week after the final MPTP injection at coordinates which was suggested by Zhuo et al. in a previous study (Zhuo et al. 2024). The control group received saline of same amount as a blank control with identical injection method. Each experimental group and the control group consisted of 6 mice.

### Animal behavior tests

The mice were taken for behavior experiments including open field test and APO rotation test at the 4th to 6th weeks, and sacrificed for autopsy analysis after 6 weeks. In the open field test, the mice were placed in the middle of an experiment case with a size of 50 × 50 × 40 cm, and the bottom of the box was divided into squares of same area when enclosed by an opaque black box. Then the black box was removed after 30 s and the behavior of mice was recorded by video for 3 min. The video images were analyzed using Image OF software (O’Hara & Co., Ltd., Japan). In the rotation-induction test, the mice from each group were injected APO (Sigma-Aldrich, United States) of 0.5 mg/kg. The number of rotations of induced PD rats within 30 min was compared with the model and blank control, and the improvement of motor function of rats in the experimental group was observed. 

### H&E and nissl staining

For H&E staining, after unfrozen with water at room temperature, the tissues were first stained with Harris hematoxylin solution (Merck, United States) for 1 min, Then the sample slides were rinsed with tap water for 15 min and placed in distilled water and 95% alcohol for 30 s, respectively. The samples were counterstained with eosin Y solution (Merck, United States) for 1 min. Finally, the slides were dehydrated and mounted with resinous mounting medium (Merck, United States).

For Nissl staining, after the samples were hydrated, the slides were placed in a cresyl violet acetate solution (Merck, United States) for 5 min. Then After rinsed in distilled water for 3 times, the slides were dehydrated with 95% alcohol and mounted with resinous mounting medium.

### Statistical analysis

All data presented in this study were analyzed with consistent values and expressed as mean ± standard deviation (SD). Data were analyzed using SPSS software (version 26.0; IBM Inc.), differences in gene expression levels were analyzed using paired t-tests, and correlations between data were analyzed using Pearson correlation analysis. *p* < significance was defined as p < 0.05.

## Results

### Isolation and Identification of OM-MSC exosomes

**Fig. 1:**
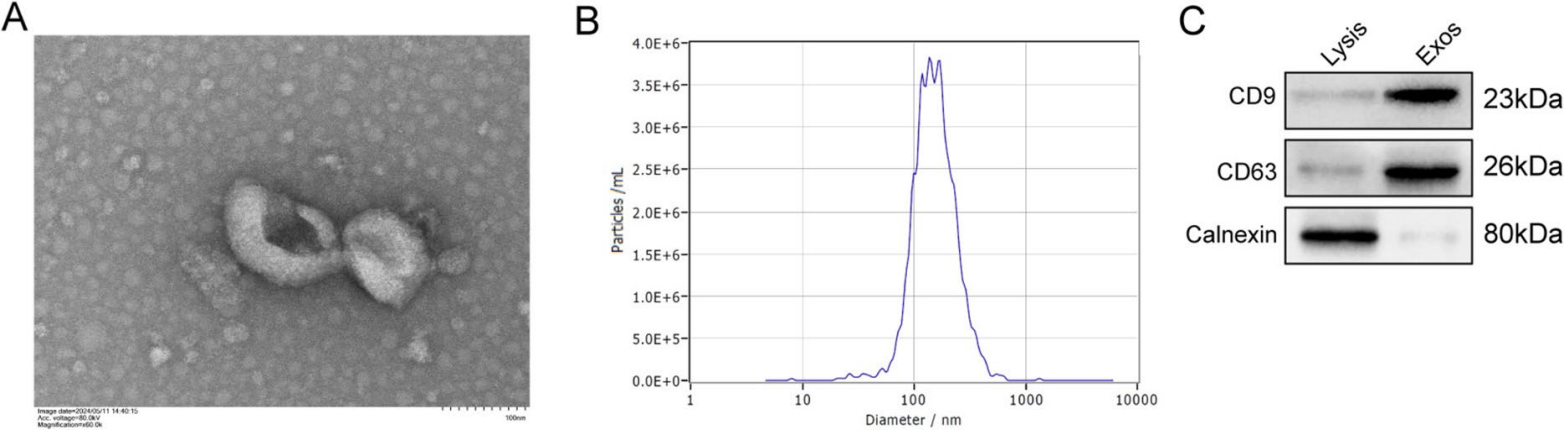
Isolation and Identification of OM-MSC Exosomes. (**A**) Analysis of sizes and morphology of the particles isolated from OM-MSCs by transmission electron microscope. (**B**) Nanoparticle tracking analysis of particles from OM-MSCs. (**C**) Western blotting analysis of CD9, CD63 and Calnexin in exosomes from OM-MScs. Data were displayed as mean ± SD.^*^p < 0.05, ^**^p < 0.01, ^***^p < 0.001

In the present study, we isolated nanoparticles from OM-MSCs. The particles were examined using transmission electron microscopy and showed classical exosome properties as double-membrane vesicles (Fig. 1A). These vesicles ranged from 100 to 140 nm, which is consistent with the characteristic size range of exosomes (Fig. 1B). Western blotting also proved that CD9 and CD63 were positive and calnexin was negative in these OM-MSC-derived vesicles (Fig. 1C). These results suggested that we successfully isolated OM-MSC-Exos.

### OM-MSC-derived exosomes relieved oxidative stress by promoting mitophagy of neurons

**Fig. 2:**
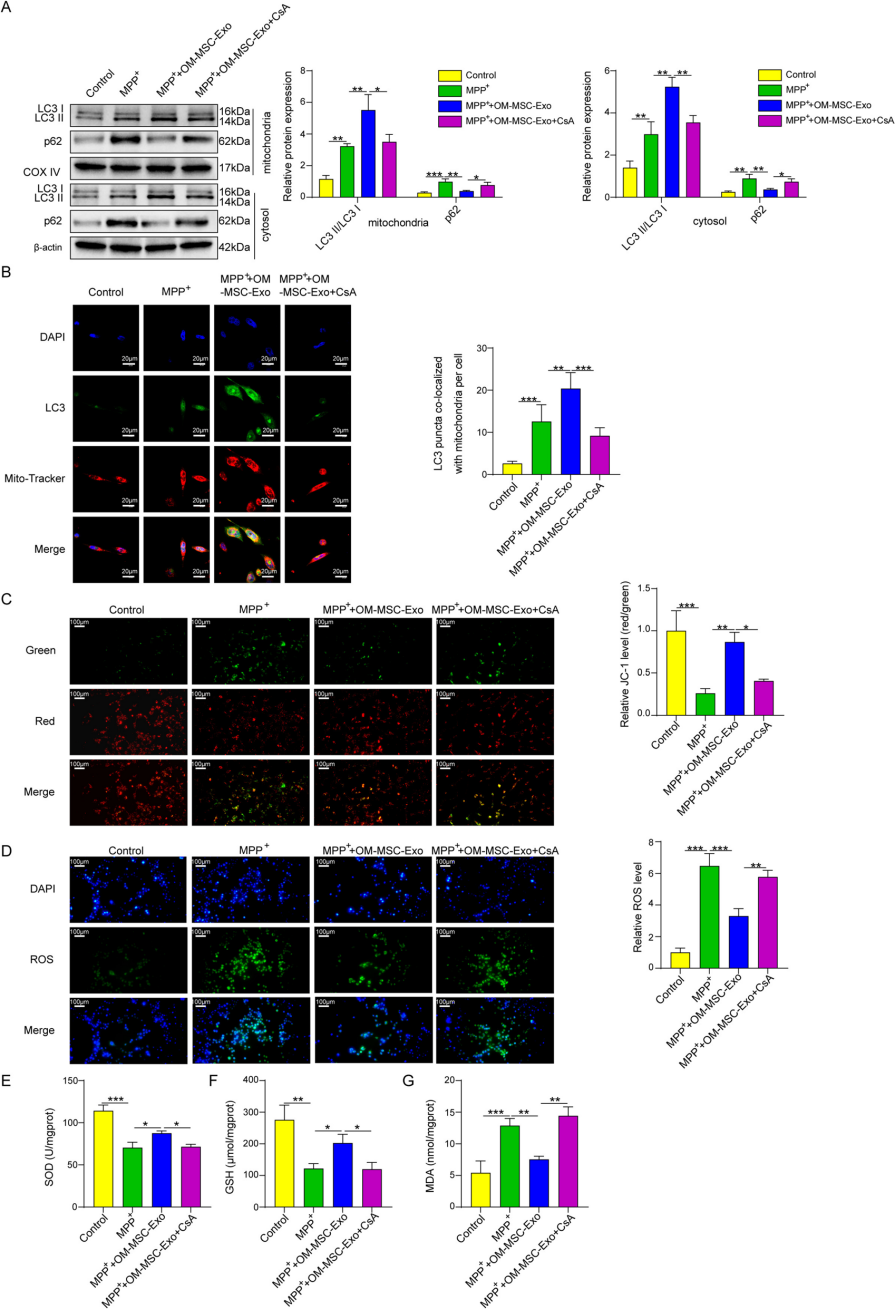
OM-MSC-derived Exosomes Relieved Oxidative stress by Promoting Mitophagy of Neurons. (**A**) Analysis of LC3, p62, COX IV expression level in mitochondria and LC3 and p62 expression level in cytoplasm of HT22 cells by Western blotting. (**B**) The LC3 level in mitochondria measured by immunofluorescence. (**C**) The Mitochondrial membrane potential detected with JC-1 fluorescent probe. (**D**) The ROS level evaluated with DCFH-DA fluorescent probe. (**E**) The SOD level in HT22 cells. (**F**) The GSH level in HT22 cells. (**G**) The MDA level in HT22 cells. Data were displayed as mean ± SD. ^*^p < 0.05, ^**^p < 0.01, ^***^p < 0.001

HT22 cells was induced in oxidative stress via MPP^+^. The cells were co-cultured with exosomes to detect the role of OM-MSC-derived exosomes in the oxidative stress of HT22 cells. In one experimental group, 10 μg of OM-MSC-derived exosomes was added, and in another experimental group, exosomes of the same amount were added together with the mitophagy inhibitor CsA. HT22 cells cultured in the same medium containing distilled water were used as controls. Western blotting and qPCR results showed that MPP^+^ significantly upregulated the expression level of LC3 in both mitochondria and cytoplasm in HT22 cells and upregulated p62 expression after 48 h of co-culture, indicating that the mitophagic flux was obstructed. Addition of OM-MSC exosomes further induced an improvement in LC3 expression while inhibiting p62 expression. However, the expression of LC3 decreased and p62 expression increased again after the addition of the mitophagy inhibitor CsA (Fig. 2A). On the other hand, The expression of LC3 was inhibited in HT22 cells by MPP + , and OM-MSC exosomes significantly upregulated LC3 levels in these cells, while CsA inhibited this reverse process (Fig. 2B). Immunofluorescence indicated that MPP^+^ significantly reduced the mitochondrial membrane potential and the amount of SOD and GSH and upregulated the amount of MDA. OM-MSC exosome treatment inhibited this trend to a large degree, while adding CsA reversed the effects of exosomes in HT22 cells, and all the reactive oxygen species were quantified based on the normalization of the control group (Fig. 2 C-G). These results demonstrated that OM-MSC exosomes ameliorated oxidative stress by promoting mitophagy in neuronal cells.

### Lnc A2M-AS1 from OM-MSC-derived exosomes induced mitophagy to ameliorate oxidative stress

**Fig. 3:**
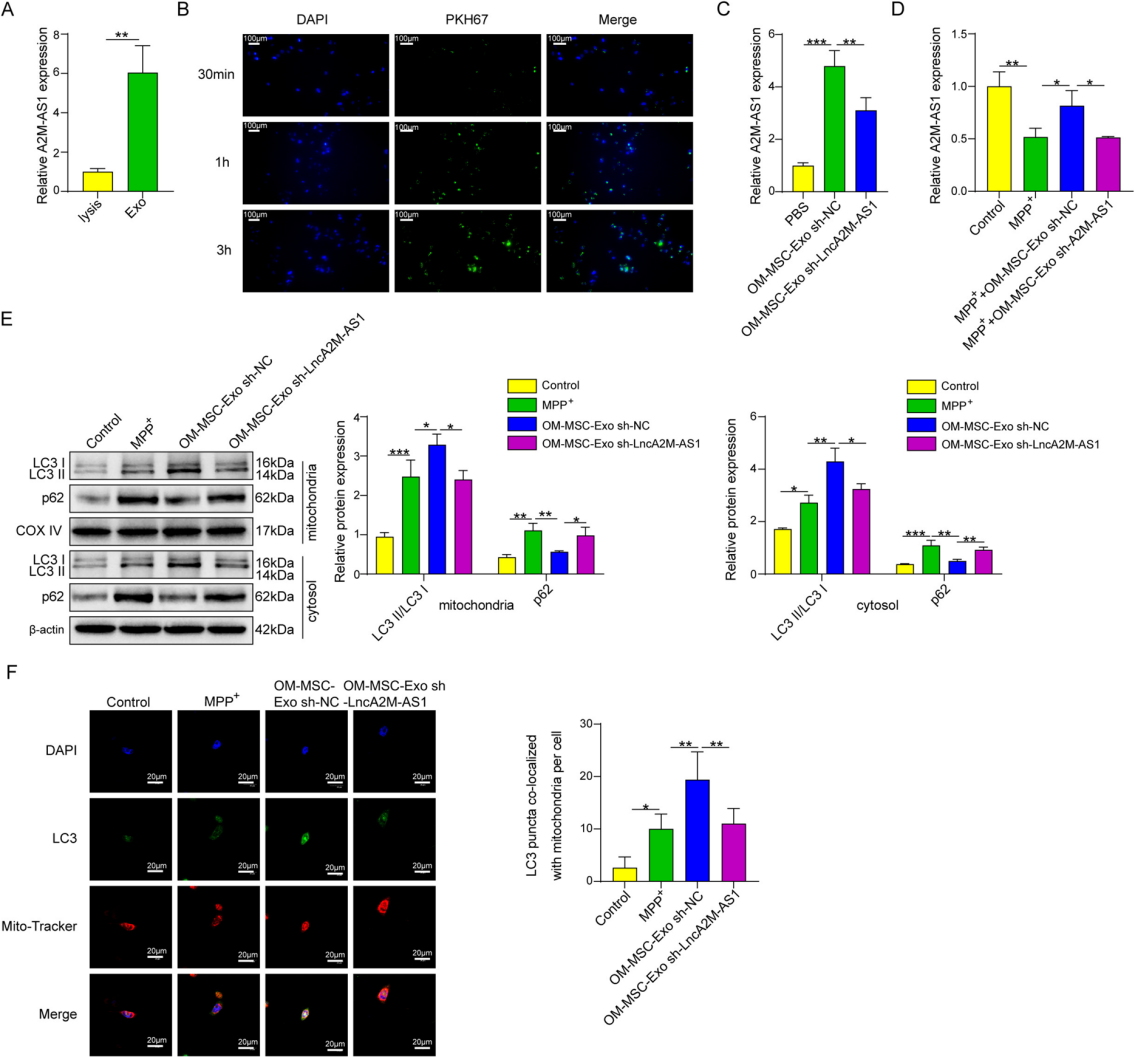

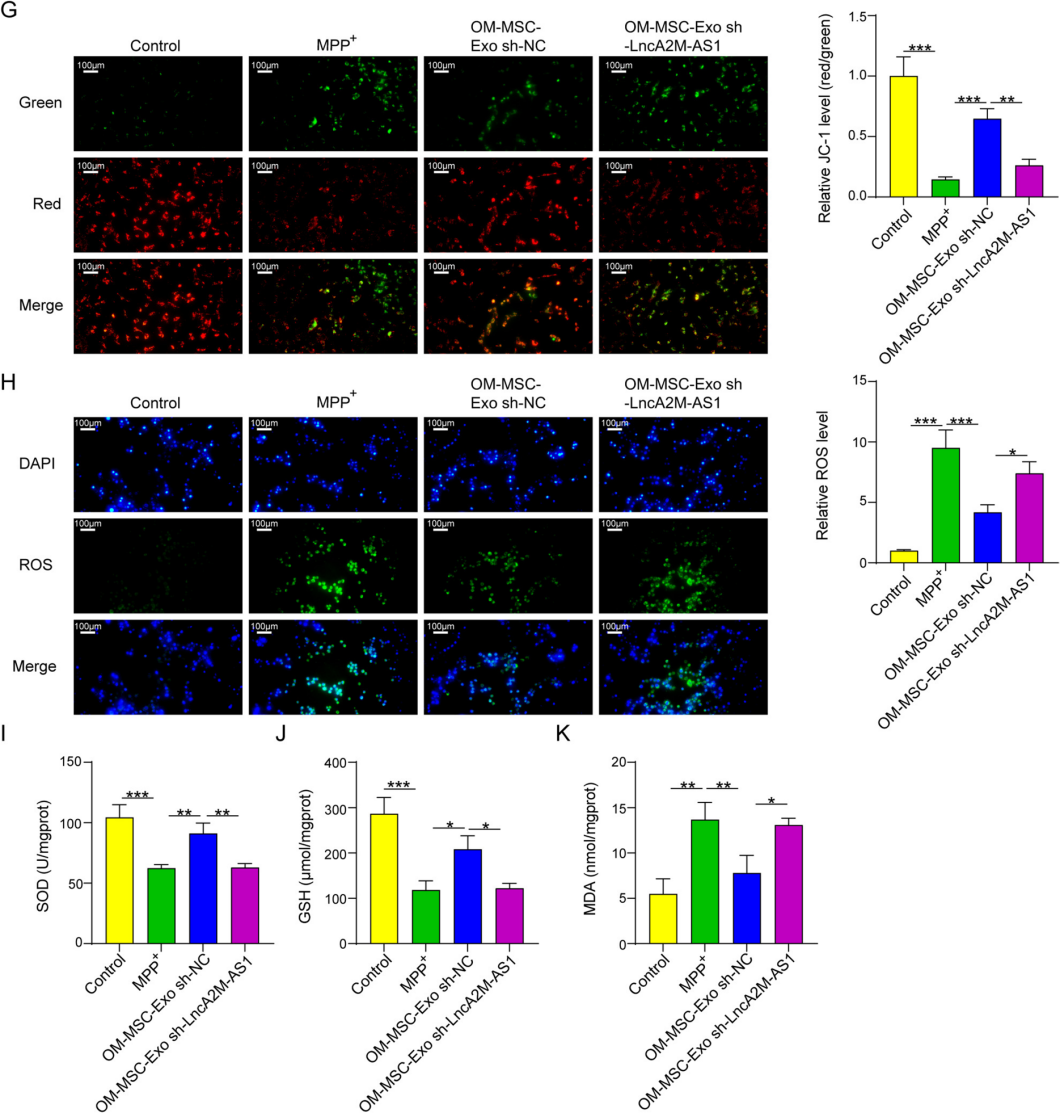
Lnc A2M-AS1 from OM-MSC-derived Exosomes Induced Mitophagy to Ameliorate Oxidative stress. (**A**) A2M-AS1 expression level in isolated OM-MSC exosomes evaluated by qPCR. (**B**) Ingestion of OM-MSC exosomes of HT22 cells evaluated by PKH67 dye. (**C**) Evaluation of the influence of OM-MSC-Exo on A2M-AS1 expression in HT22 cells by qPCR after A2M-AS1 knockdown. (**D**) Evaluation of the influence on A2M-AS1 from OM-MSC exosomes by qPCR. (**E**) Analysis of LC3, p62, COX IV expression level in mitochondria and LC3 and p62 expression level in cytoplasm of HT22 cells by Western blotting. (**F**) The LC3 level in mitochondria was measured by immunofluorescence. (**G**) The mitochondrial membrane potential was evaluated with JC-1 fluorescent probe. (**H**) The ROS levels in HT22 cells were detected with DCFH-DA fluorescent probe. (**I**) The SOD level in HT22 cells. (**J**) The GSH level in HT22 cells. (K) The MDA level in HT22 cells. Data were displayed as mean ± SD. ^*^p < 0.05, ^**^p < 0.01, ^***^p < 0.001

The expression level of long noncoding RNA(lncRNA) A2M-AS1 in exosomes isolated from OM-MSC was measured by qPCR. Compared with cell lysates, lncRNA A2M-AS1 was much higher in the isolated exosomes in the supernatant (Fig. 3A). HT22 cells were co-cultured with PK67 labeled OM-MSC-derived exosomes to verify the ingestion process of exosomes, and cells cultured with the same amount of PBS were used as the control groups. The cells were observed at the initial time point and at 1 and 3 h. The results indicated that the fluorescent signal increased gradually from the initial state, and after 3 h of incubation, the PK67 signal from the OM-MSC exosomes was observed in most of the cells (Fig. 3B). We also observed the influence of OM-MSC-derived exosomes on A2M-AS1 expression in HT22 cells. qPCR results indicated that these exosomes could significantly upregulate the expression of this lncRNA in HT22 cells. Furthermore, A2M-AS1 expression in HT22 cells significantly declined after A2M-AS1 knockdown in exosomes (Fig. 3C and D). In the present study, western blotting results proved that OM-MSC-derived exosomes greatly upregulated LC3 expression and downregulated p62 expression in both the mitochondria and cytoplasm of MPP^+^-induced HT22 cells. This study also confirmed that A2M-AS1 knockdown in OM-MSC-derived exosomes could reverse this effect of exosomes on mitophagy-related proteins (Fig. 3E). We also observed LC3 levels in mitochondria using immunofluorescence technology, and the result showed that OM-MSC-derived exosomes could considerably upregulate LC3 expression in mitochondria of MPP^+^ induced neurons, and knock down A2M-AS1 in OM-MSC-derived exosomes could reverse this upregulation effect from this exosome on mitochondria LC3(Fig. 3F). Other results showed that OM-MSC-derived exosomes significantly increased SOD and GSH levels in MPP + -induced neurons and reduced the levels of ROS and MDA, as well as the membrane potential of mitochondria, indicating that these exosomes could *reduce* oxidative stress by enhancing mitophagy. However, A2M-AS1 knockdown in these exosomes partially reversed the effects of OM-MSC-derived exosomes on oxidative stress (Fig. 3G-K). Thus, the above results demonstrated that lncRNA A2M-AS1 from OM-MSC-derived exosomes could ameliorate oxidative stress by inducing mitophagy.

### Lnc A2M-AS1 from OM-MSC-derived exosomes induced mitophagy through tp53inp1 to ameliorate oxidative stress

**Fig. 4:**
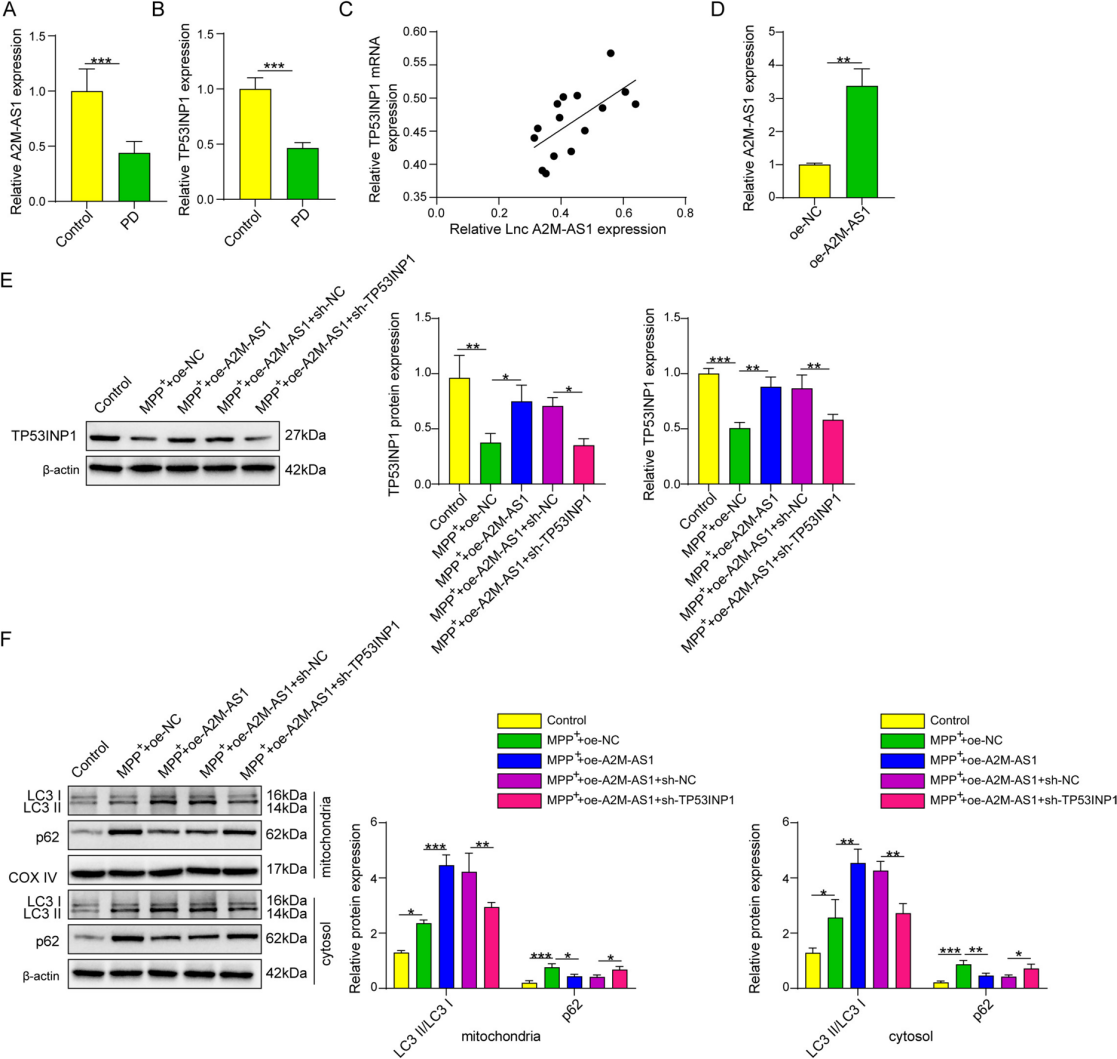

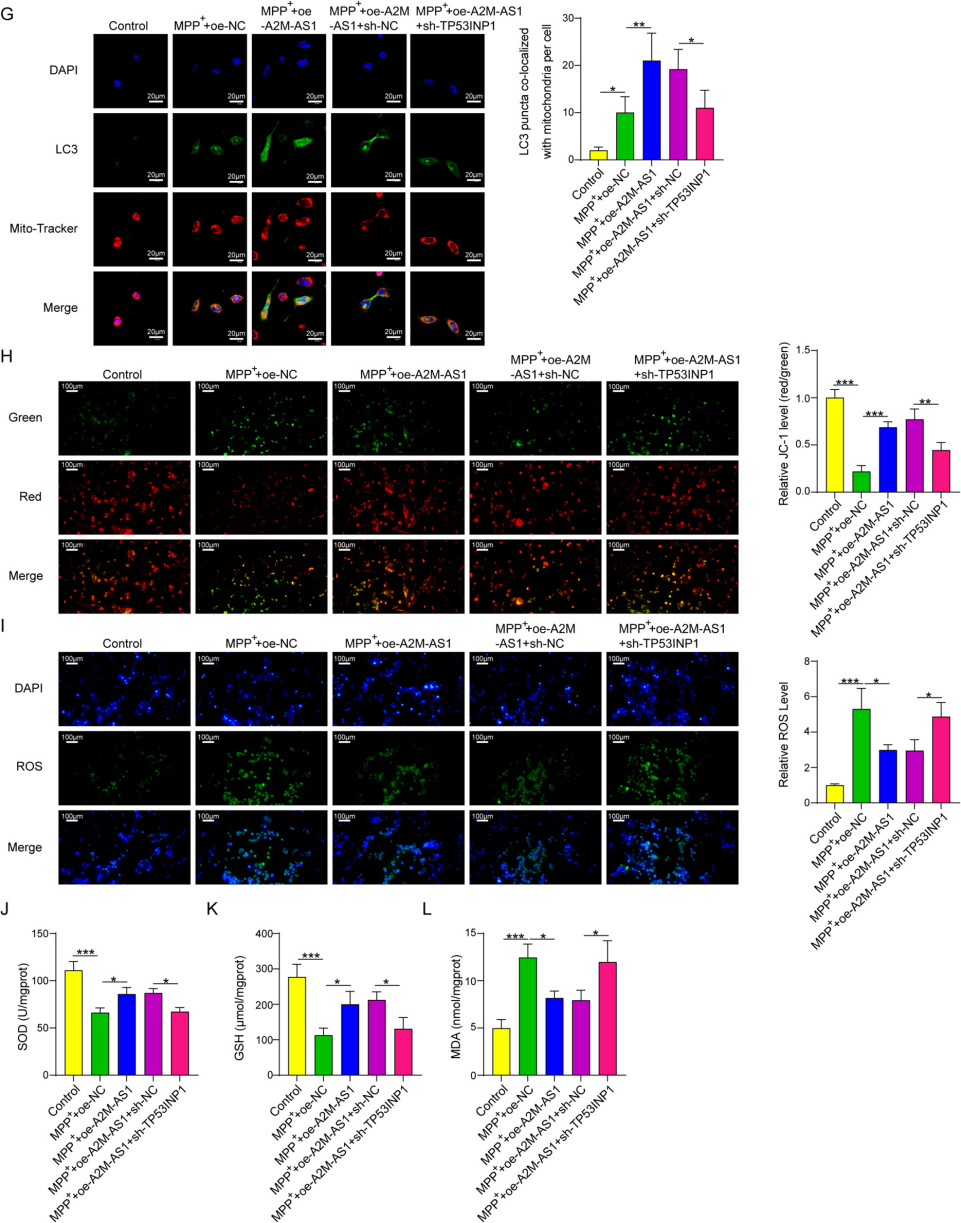
Lnc A2M-AS1 from OM-MSC-derived Exosomes Induced Mitophagy through TP53INP1to Ameliorate Oxidative Stress. (**A**) A2M-AS1 expression level in the serum of normal control subjects and PD patient measured by qPCR. (**B**) TP53INP1 expression level in serum of normal control subjects and PD patient measured by qPCR. (**C**) Analysis of the expression correlation between A2M-AS1 and TP53INP1 in PD based on data in A and B. (**D**) Influence of overexpression-A2M-AS1 plasmid (oe-A2M-AS1) to the expression of A2M-AS1 evaluated by qPCR. (**E**) Influence of oe-A2M-AS1 and short hairpin TP53INP1 (shTP53INP1) to the expression of TP53INP1 evaluated by qPCR and Western blotting. (**F**) Analysis of LC3, p62, COX IV expression level in mitochondria and LC3 and p62 expression level in cytoplasm of HT22 cells by Western blotting. (**G**) The LC3 level in mitochondria was measured by immunofluorescence. (**H**) The mitochondrial membrane potential was evaluated with JC-1 fluorescent probe. (**I**) The ROS level detected with DCFH-DA fluorescent probe. (**J**) The SOD level in HT22 cells. (**K**) The GSH level in HT22 cells. (L) The MDA level in HT22 cells. Data were displayed as mean ± SD. ^*^p < 0.05, ^**^p < 0.01, ^***^p < 0.001

The bioinformation prediction based on starBase showed that Lnc A2M-AS1 could bind with IGFBP1, and IGF2BP1 could bind with the mRNA of TP53INP1. The expressions of A2M-AS1 and TP53INP1 in tissues and serum from patients with PD was evaluated using qPCR. The results indicated that both genes had low expression levels in the brain tissue and serum of PD patients, and a positive correlation existed between the expression levels of A2M-AS1 and TP53INP1(Fig. 4A-C). This study also showed that oe-A2M-AS1 could upregulate the expression of its target gene (Fig. 4D), and oe-A2M-AS1 significantly upregulated TP53INP1 expression in MPP^+^-induced neurons, while knockdown of TP53INP1 partially reversed this effect (Fig. 4E). Furthermore, oe-A2M-AS1 could increase LC3 and reduce p62 expression in mitochondria and plasma in MPP^+^ induced neurons, and knocking down TP53INP1 could reverse this influence of oe-A2M-AS1 on these mitophagy related proteins (Fig. 4F). This upregulation effect of oe-A2M-AS1 on LC3 expression in MPP^+^-induced neurons and the reverse effect of TP53INP1 knockdown were verified by immunofluorescence analysis (Fig. 4G). Mitochondrial membrane potential was evaluated using JC-1 probes, and the levels of GSH, SOD, MDA, and ROS were measured. The results showed that A2M-AS1 overexpression reduced oxidative stress in MPP^+^-induced HT22 cells by inducing mitophagy through TP53INP1. Furthermore, A2M-AS1 increased the levels of GSH and SOD in HT22 cells and reduced the levels of ROS and MDA. Downregulation of TP53INP1 also reversed this effect (Fig. 4H-L). Thus, we indicated that lncRNA A2M-AS1 could ameliorate oxidative stress by promoting mitophagy through TP53INP1.

### Lnc A2M-AS1 mediates expression of TP53INP1 by targeting at IGF2BP1

**Fig. 5:**
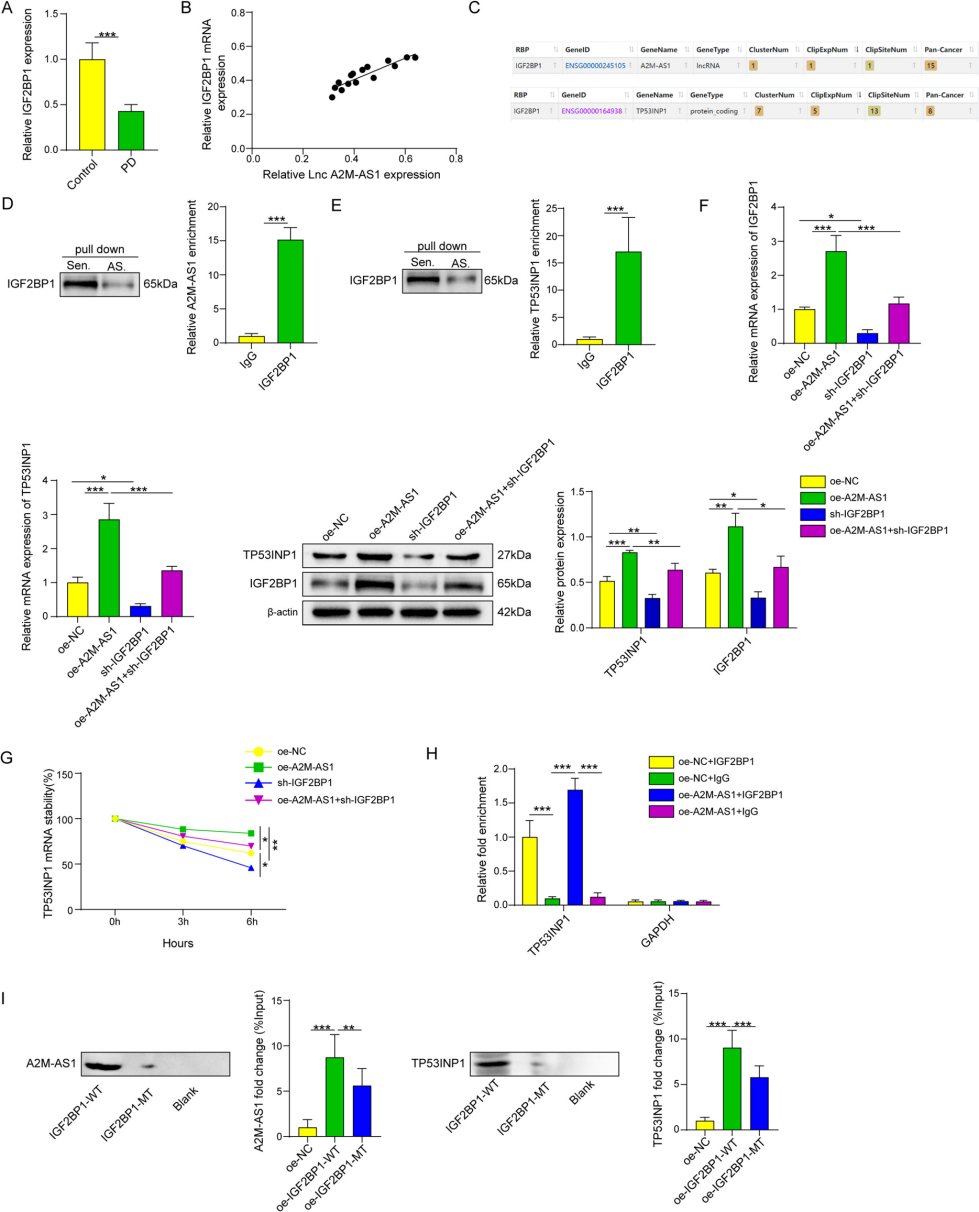
Lnc A2M-AS1 Mediates Expression of TP53INP1 by Targeting at IGF2BP1. (**A**) IGF2BP1 expression level in serum of normal control subjects and PD patient measured by qPCR. (**B**) Analysis of the expression correlation relationships between A2M-AS1 and IGF2BP1 in PD. (**C**) The binding between A2M-AS1 and IGF2BP1 verified by RNA pull-down and RIP assays. (**D**) The binding between TP53INP1 and IGF2BP1 verified by RNA pull-down and RIP assays. (**E**) Expression of IGF2BP1 evaluated by qPCR and Western Blotting. (**F**) Expression of TP53INP1 evaluated by qPCR and Western Blotting. (**G**) Detection of the influence of A2M-AS1/IGF2BP1 on TP53INP1 mRNA stability by qPCR. Data were displayed as mean ± SD. ^*^p < 0.05, ^**^p < 0.01, ^***^p < 0.001

We found that A2M-AS1 and IGF2BP1 have relatively low expression levels in PD patients compared with normal individuals, and the expression of A2M-AS1 was positively correlated with IGF2BP1(Fig. 5A-B). The RNA pull-down results indicated that both TP53INP1 and A2M-AS1 were captured by IGF2BP1. Bioinformation analysis based on starBase database was conducted in this study, and the results showed that Lnc A2M-AS1 could bind with IGFBP1, and IGF2BP1 could bind with the mRNA of TP53INP1. Those results suggested that Lnc A2M-AS1 could mediate the stability and expression of TP53INP1 mRNA via its interaction with IGF2BP1 protein (Fig. 5C). The results of RNA pull down/RIP analysis suggested that A2M-AS1 and TP53INP1 could both bind with IgF2BP1, and Results compared with IgG also showed enrichment of A2M-AS1 and TP53INP1 in pellets of IGF2BP1 antibodies, indicating that IGF2BP1 could bind with those molecules (Fig. 5D-E). Furthermore, modifying the expression of A2M-AS1 showed that oe-A2M-AS1 significantly upregulated the expression of IGF2BP1 and TP53INP1 and enhanced the stability of TP53INP1 mRNA. Application of sh-IGF2BP1 significantly reduced the expression of IGF2BP1 and TP53INP1 and weakened the stability of TP53INP1 mRNA. Moreover, sh-IGF2BP1 partially reversed the stimulatory effect of lncRNA A2M-AS1 (Fig. 5 F-G). RIP analysis results also demonstrated that oe-A2M-AS1promoted the binding level between IGF2BP1 and TP53INP1 (Fig. 5H). We also used mutation analysis after the prediction for the binding sites between A2M-AS1 and TP53INP1, and A2M-AS1 and IGF2BP1. The mutation analysis showed that mutations on these sites could inhibit the interactions between the molecules above(Fig. 5H). In addition, the RNA pull-down analysis also validated that LncA2M-AS1 and TP53INP1 could both bind with IGF2BP1 wild type. These results indicate that lncRNA A2M-AS1 mediates the expression of TP53INP1 by binding to IGF2BP1.

### OM-MSC exosome derived Lnc A2M-AS1 Improves PD condition by mediating mitophagy to reduce oxidative stress effects

**Fig. 6:**
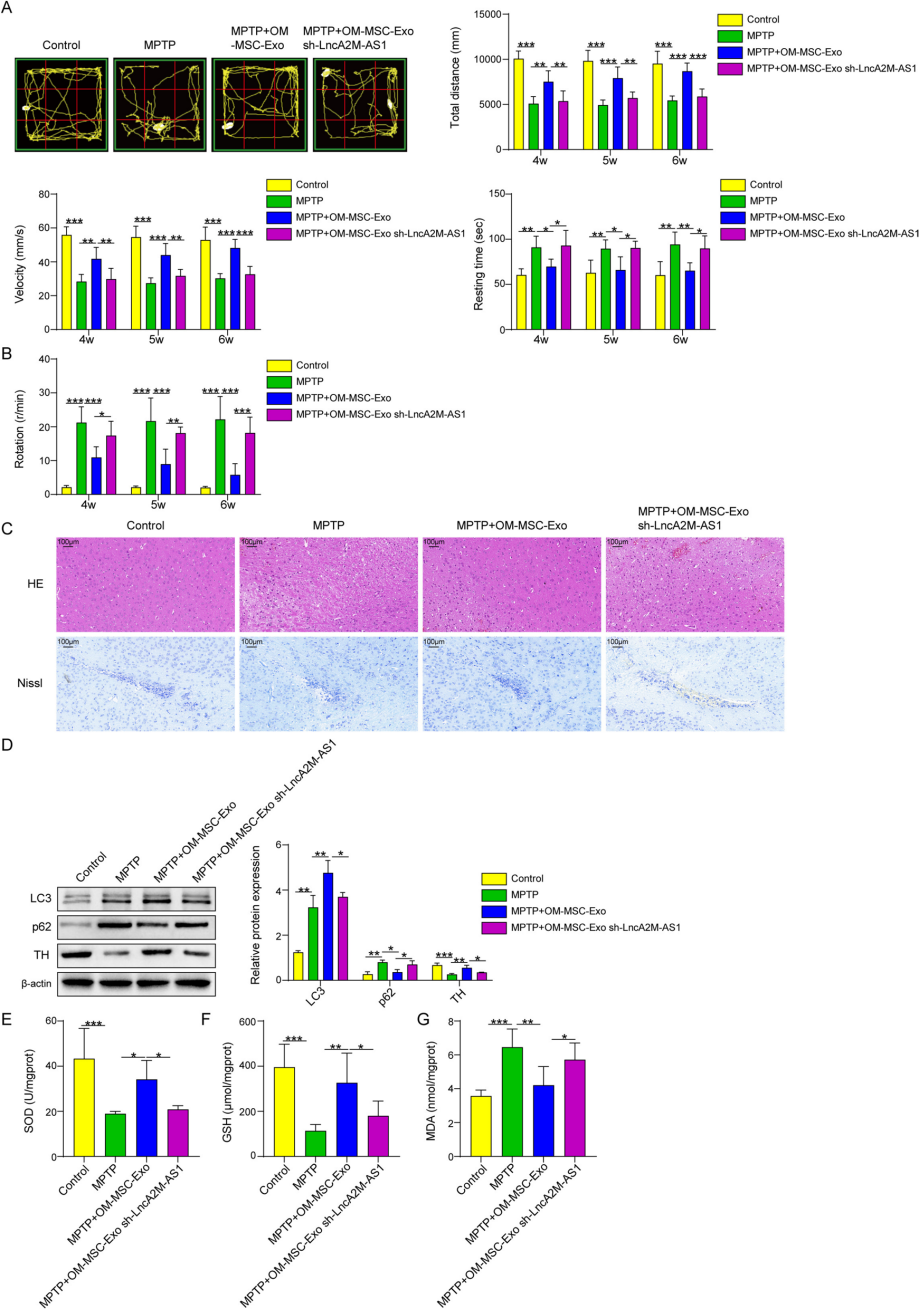
OM-MSC Exosome Derived Lnc A2M-AS1 Improves PD Condition by Mediating Mitophagy to Reduce Oxidative Stress Effects. (**A**) Total distance moved, speed of movement and rest time during a period of 10 min in the 4th, 5th and 6th week in open field test. (**B**) ipsilateral rotation in APO rotation test after 4 weeks. (**C**) HE staining (top) and Nissl staining (bottom) of mice brain tissues. (**D**) Expression of TH, LC3 and p62 evaluated by Western blotting. (**E**) The SOD level in mice brain tissue. (**F**) The GSH level in mice brain tissue. (**G**) The MDA level in mice brain tissue. Data were displayed as mean ± SD. ^*^p < 0.05, ^**^p < 0.01, ^***^p < 0.001

Animal behavior tests were also applied in this study to verify the role played by Lnc A2M-AS1 in vivo. PD mice treated with OM-MSC-Exosomes showed longer travel distance and faster movement speed altogether with shorter rest time in open field testing. The APO rotation scores also declined significantly (Fig. 6 A-B). However, this effect on mice in the above behavior tests could be reversed partially by treatment with OM-MSC exosomes which were knocked down A2M-AS1. Compared with typical neuron structures from normal mice, mice treated with MPTP showed a decrease in the number of neurons and cells with Nissl bodies in the substantia nigra tissue. Nissl staining showed that the mice from the group treated together with MPTP and OM-MSC exosome had a significant improvement of the Nissl body positive cells. However, knocking down A2M-AS1 in OM-MSC exosomes would reverse this improvement effect on neurons from PD mice. **(**Fig. 6C**).** Western blotting results proved that A2M-AS1 could increase the ratio between LC3II and LC3 I, and TH and reduce p62 expression in mice treated with MPTP in substantia nigra tissue. But treatments with A2M-AS1 knocked-down OM-MSC-derived exosomes would also reverse the effect on the expressions of the proteins above (Fig. 6D). As for the antioxidation substances, OM-MSC-derived exosomes could significantly increase the amount of SOD and GSH and decrease the level of MDA in mice substantia nigra treated with MPTP, which also showed the oxidation stress level in the substantia nigra tissue(Fig. 6E-G). This effect could also be partially reversed by knocking down A2M-AS1 in OM-MSC exosomes. These results proved that OM-MSC-derived Lnc A2M-AS1 could improve PD conditions by mediating mitophagy to reduce oxidative stress effects.

## Discussion

Recently, studies on treatments against various diseases using stem cells and stem cell-derived exosomes have received appreciable results, especially regarding neurodegenerative diseases (Mendes-Pinheiro et al. 2019; D'Egidio et al. 2024; Pedrosa et al. xxxx). As a safe and convenient source of autologous stem cells, the use of OM-MSCs to treat neurological diseases has attracted great research interest. Current research on these cells mainly focuses on neuropsychiatric diseases, such as schizophrenia and refractory epilepsy (Zhong et al. 2023; Liu et al. 2023). We believe that this kind of cell holds credible potential as a candidate for the treatment of neurodegenerative diseases. In the present study, we studied the interaction between Lnc A2M-AS1, which is one of the exosomes from OM-MSC, and HT22 cells under oxidative stress induced by MPP^+^ and analyzed the changes in the expression of various molecules in such cells. This study demonstrated that lncRNA A2M-AS1 reduces oxidative stress and improves Parkinson's disease by regulating TP53INP1-mediated mitophagy through interaction with IGF2BP1 by combining molecular results and results of clinical samples and animal experiments both in vivo and in vitro.

In the present study, we successfully isolated exosomes from OM-MSCs. We confirmed that such exosomes could increase the intracellular antioxidant content and alleviate oxidation by inducing mitophagy in HT22 cells that enter the oxidative stress state through MPP^+^ stress. In addition, we also demonstrated that this process was from lncRNA A2M-AS1 inside the exosomes based on qPCR analysis, immunofluorescence, and gene knockdown results. Previous studies have indicated that OM-MSC have enormous potential as a possible treatment for Alzheimer’s disease (Hong et al. 2022), and Simorgh et al. and Li et al. also showed that these kinds of cells might play an important role in the treatment of PD (Simorgh et al. 2019; Lee et al. 2024). As an important stem cell, Xun et al. pointed out that OM-MSC have a relatively high ability to produce exosomes, which have been shown to play important roles in multiple processes of tissue and neuron fixing (Xun et al. 2020). Mitophagy is widely considered an important repair mechanism for oxidative stress injuries. Okarmus et al. indicated that promoting mitophagy can reduce oxidative stress damage in neurons and may therefore serve as a potential therapy for Parkinson’s disease (Okarmus et al. 2024). In addition, Yu et al. revealed that lncRNA A2M-AS1 effectively relieved oxidative stress injuries from ischemia reperfusion in cardiomyocytes (Yu et al. 2023). Previous studies have also shown that this Lnc RNA plays important roles in neurodegenerative diseases, such as Alzheimer’s disease (Li et al. 2022). Therefore, OM-MSC-derived Lnc A2M-AS1 is considered a candidate therapeutic agent against PD.

Multiple studies have demonstrated that lncRNAs can regulate the expression of various downstream proteins by binding to their target RNA-binding proteins (Wang et al. 2024). As one of the important exosome Lnc RNA from OM-MSCs, A2M-AS1 binds to multiple proteins, such as IGFBP1, to regulate a series of important physiological processes and gene expressions (Qiu et al. 2022). Previous studies have demonstrated that TP53INP1 plays a role in the apoptosis of damaged cells. Furthermore, Dinh et al. showed that TP53INP1 is involved in regulating oxidative stress processes (Seillier et al. 2015). A study by Seillier et al. showed that a lack of TP53INP1 drives the occurrence and development of a series of metabolic syndromes under oxidative stress (Dinh et al. 2021). In the present study, we demonstrated that knockdown of TP53INP1 could reverse the promotion of mitophagy by A2M-AS1 in HT22 cells to prevent this process of improving oxidative stress, consistent with previous studies in non-neuronal cells (Sancho et al. 2012; Seillier et al. 2012). Besides, it analysis revealed that IGF2BP1 was the target of A2M-AS1 to regulate TP53INP1 expression in order to mediate oxidative stress. Therefore, it would be a potential direction to find PD medication solutions through the mediation of mitophagy to relieve oxidative stress and recover the function of dopaminergic neurons by targeting such molecules.

It has been proven that lncRNAs can exist in exosomes in considerable amounts, and exosomes actively transfer bioactive molecules between neurons (Lizarraga-Valderrama and Sheridan 2021; Rufino-Ramos et al. 2017). Wang et al. also provided evidence that exosomes participate in the maintenance of function, differentiation, and apoptosis of neuronal complexes in PD (Wang et al. 2022). For example, Lnc MKRN2-42 is related to the pathophysiology of PD, and Elkouris et al. discovered that exosomal Lnc RNA were closely linked with PD pathogenesis (Wang et al. 2020; Elkouris et al. 2019). In this study, we found that lncRNA A2M-AS1 from OM-MSC exosomes could effectively improve PD symptoms through animal behavior tests. Our observation of tissue samples from PD mice also confirmed that lncRNA A2M-AS1 could improve the morphological structure of neurons in PD animals and upregulate antioxidant molecules such as SOD and GSH in neurons, which is also consistent with previous reports that exosomes are involved in regulating the oxidative stress process in PD (Wang et al. 2022).

This study had a few limitations. For instance, in behavioral testing, mice may not fully mimic the effects of these exosomes in human subjects with PD. Therefore, animals closer to humans are required for further behavioral testing. Furthermore, functional imaging is required in future studies to improve the analysis of the impact of exosomes on neurological function in living PD animals.

## Conclusions

In conclusion, this study revealed that lncRNA A2M-AS1 delivered through OM-MSC-derived exosomes suppressed oxidative stress in PD through the IGF2BP1/TP53INP1 axis by mediating mitophagy. Through this pathway, OM-MSC-derived exosomes could improve clinical and behavioral results in mice with PD and could be a potential candidate for new therapeutic methods for PD.

## Data Availability

The datasets used or analyzed during the current study are available from the corresponding author upon reasonable request.

## Abbreviations

OM-MSC: Olfactory Mucosal Mesenchymal Stem Cell
OM-MSC-Exo: Exosome Derived from Olfactory Mucosal Mesenchymal Stem Cells
LncRNA/Lnc: Long Noncoding RNA
A2M-AS1: Long Noncoding RNA A2M-AS1
IGF2BP1: Insulin-like Growth Factor 2 mRNA Binding Protein 1
TP53INP1: Tumor Protein p53 Inducible Nuclear Protein 1
MPP +: 1-Methyl-4-phenylpyridinium
MPTP: 1-Methyl-4-phenyl-1,2,3,6-tetrahydropyridine
qPCR: Quantitative Polymerase Chain Reaction
RIP: RNA Immunoprecipitation
ActD: Actinomycin D
LC3: Microtubule-associated Protein 1 Light Chain 3
p62: Sequestosome 1 (Autophagy Marker)
SOD: Superoxide Dismutase
GSH: Glutathione
MDA: Malondialdehyde
ROS: Reactive Oxygen Species
TH: Tyrosine Hydroxylase
COX IV: Cytochrome c Oxidase Subunit IV
PBS: Phosphate Buffer Saline
